# Unusual dimensionality effects and surface charge density in 2D Mg(OH)_2_

**DOI:** 10.1038/srep20525

**Published:** 2016-02-05

**Authors:** Aslihan Suslu, Kedi Wu, Hasan Sahin, Bin Chen, Sijie Yang, Hui Cai, Toshihiro Aoki, Seyda Horzum, Jun Kang, Francois M. Peeters, Sefaattin Tongay

**Affiliations:** 1 School for Engineering of Matter, Transport and Energy, Arizona State University, Tempe, AZ, 85287, USA; 2 Department of Physics, University of Antwerp, Campus Groenenborger, Groenenborgerlaan 171, 2020, Antwerp, Belgium; 3 LeRoy Eyring Center for Solid State Science, Arizona State University, Tempe, AZ 85287, USA

## Abstract

We present two-dimensional Mg(OH)_2_ sheets and their vertical heterojunctions with CVD-MoS_2_ for the first time as flexible 2D insulators with anomalous lattice vibration and chemical and physical properties. New hydrothermal crystal growth technique enabled isolation of environmentally stable monolayer Mg(OH)_2_ sheets. Raman spectroscopy and vibrational calculations reveal that the lattice vibrations of Mg(OH)_2_ have fundamentally different signature peaks and dimensionality effects compared to other 2D material systems known to date. Sub-wavelength electron energy-loss spectroscopy measurements and theoretical calculations show that Mg(OH)_2_ is a 6 eV direct-gap insulator in 2D, and its optical band gap displays strong band renormalization effects from monolayer to bulk, marking the first experimental confirmation of confinement effects in 2D insulators. Interestingly, 2D-Mg(OH)_2_ sheets possess rather strong surface polarization (charge) effects which is in contrast to electrically neutral h-BN materials. Using 2D-Mg(OH)_2_ sheets together with CVD-MoS_2_ in the vertical stacking shows that a strong change transfer occurs from *n*-doped CVD-MoS_2_ sheets to Mg(OH)_2_, naturally depleting the semiconductor, pushing towards intrinsic doping limit and enhancing overall optical performance of 2D semiconductors. Results not only establish unusual confinement effects in 2D-Mg(OH)_2_, but also offer novel 2D-insulating material with unique physical, vibrational, and chemical properties for potential applications in flexible optoelectronics.

Layered crystals at the quantum confinement (2D) limit are emerging as an important class of materials for information and energy conversion technologies^1^,^2^. Especially after demonstration and synthesis of 2D materials with finite band gaps, such as h-BN and members of the semiconducting transition metal dichalcogenides (sTMDs) family^3^,^4^,^5^, a large number of proof-of-concept devices has been realized^1^,^6^. In particular, sTMDs have shown great potential in optoelectronics applications owing to their unusual properties in the quantum confinement limit^7^,^8^,^9^,^10^,^11^. However, unlike the vast number of existing conventional materials where their gap values almost continuously cover from near infrared (NIR) to deep UV, the full potential of these 2D materials is mostly limited by their energy gaps, band alignment properties, and, with respect to 3D materials, offers a relatively small number of materials to choose from.

Here, we experimentally and theoretically report on the physical, chemical, and lattice vibrations of 2D Mg(OH)_2_, a member of alkaline metal hydroxides (AMHs), for the first time. By modified Mg(OH)_2_ hydrothermal synthesis technique, we successfully isolated 2D Mg(OH)_2_ from highly crystalline, large domain size, and layered Mg(OH)_2_ crystals. Raman spectroscopy measurements on Mg(OH)_2_ show that the lattice vibration characteristics and their dimensionality dependence are in stark contrast to other 2D materials known to date. Instead of first-order Raman peaks, the lattice vibration characteristics of 2D Mg(OH)_2_ are mostly dominated by two hydroxyl (–OH) group in-phase and out-of-phase vibration modes (A_1g_^OH(1,2)^) which appear at 3642 and 3651 cm^−1^. A_1g_^OH^ peak position and shape strongly depend on the interaction between –OH groups in the adjacent layers and, in 2D, Raman spectrum reduces to out-of-plane mode. Nanoscale sub-wavelength electron energy-loss spectroscopy (nano-EELS) measurements correlate with the electronic structure to the dimensionality of the Mg(OH)_2_, showing the band renormalization effects on 2D insulating materials. Owing to the strong polarization at the AMHs surface (induced by the hydroxyl group), vertical stacking of Mg(OH)_2_/MoS_2_ enables tuning the MoS_2_ properties as evidenced by much enhanced photoluminescence (PL) emission and shifted PL peak position. Overall results expand the library of 2D materials towards insulating counter-parts, offering a new class of 2D insulating materials with unique physical, chemical, and vibrational properties.

## Structure, crystal growth, and 2D Mg(OH)_2_ sheets

**Figure 1:**
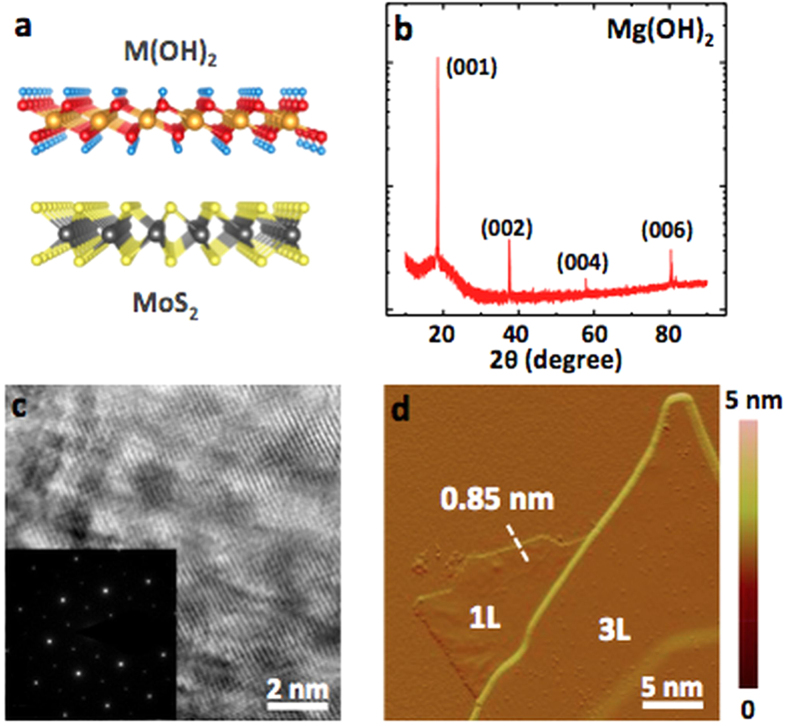
AMHs crystal structure and characterization (**a**) Structural comparison between MoS_2_, a member of TMDCs and Mg(OH)_2_ from AMHs family. (**b)** XRD pattern taken directly from Mg(OH)_2_ crystals. (**c)** Transmission electron microscopy image and SAED pattern taken from synthesized crystals displaying the crystallinity and 1T lattice structure. **(d)** Atomic force microscopy images taken from monolayer and few-layer Mg(OH)_2_.

Alkaline earth metal hydroxides are a family of layered crystals with the general formula M(OH)_2_, where M is alkaline metal from group IIA elements such as Ca and Mg^12^,^13^. Similar to transition metal dichalcogenides (TMDs), alkaline hydroxides belong to the P3m1 (Hermann-Mauguin notation) space group, except the unit cell consists of one alkaline metal group element (Ca, Mg) symmetrically bonded to two hydroxide groups in the *T*-phase as opposed to observed *H*-phase in group VI TMDs (e.g. MoS_2_, WS_2_) as shown in Fig. 1a.

Previously, AMHs crystals were synthesized by hydrothermal growth routes in small autoclave reactors in nanoparticle or micro-sized crystallite form, but small, single-domain size and poor control over their growth dynamics prevented achieving 2D- and few-layer (quasi-2D) AMHs sheets. To synthesize larger size and highly crystalline Mg(OH)_2_, alkaline metal nitrates, Mg(NO_3_)_2_ were first heated to 125–140 °C for six hours in a stainless steel autoclave (250 mL), immediately reacted with ammonia (NH_3_) for a week at 5–10 MPa^13^,^14^,^15^, and slowly cooled to room temperature at controlled rates after six days. Typical synthesis process yields ~1 mm up to cm-sized crystals dispersed in the bi-product solution. Materials were finally filtered using micropore filters and washed by DI water. Sharp (001) peaks located at multiples of 18.55° (Fig. 1b), associated with the Bragg reflections from layers stacked in the c-axis direction, confirm the crystallinity of the materials. The lattice spacing is estimated around ~4.86 A°, which is close to the values calculated by our DFT calculations (~4.95 A°).

Transmission electron microscopy (TEM) image and selected area electron diffraction (SAED) pattern also confirm high crystallinity of the synthesized materials as shown in Fig. 1c for Mg(OH)_2_ where the SAED pattern displays a hexagonal pattern expected from tetragonal distorted AMHs monolayers with three-fold symmetry. Here, we note that the monolayers quickly decomposed under an 80 keV e-beam, possibly due to the low melting point of Mg(OH)_2_, preventing us from capturing high resolution TEM images.

We isolated monolayer Mg(OH)_2_ from synthesized bulk crystals by mechanical exfoliation onto piranha cleaned 285 nm thermal SiO_2_/Si substrates for improved contrast. Resulting monolayers were visible under the optical microscope, but appeared faint or dull compared to MoS_2_ on 90 nm SiO_2_. Contact mode atomic force microscopy (AFM) measurements show that each Mg(OH)_2_ layer is ~0.85–0.95 nm in thickness (Fig. 1d).

Even though Mg(OH)_2_ quickly becomes unstable under e-beam irradiation due to relatively weak bonds lost by collusion with electrons, the monolayer is stable in ambient conditions as determined by AFM and Raman measurements: the surface morphology, smoothness, and Raman spectrum show no sign of surface degradation within a three-month timeframe and are superior to metastable or unstable 2D materials such as silicene^16^,^17^,^18^, black phosphorus^19^, MoTe_2_^20^, and GaTe. Prolonged environmental stability of Mg(OH)_2_ is most likely related to strongly bonded hydrogen and oxygen atoms in –OH groups creating chemically passivated surfaces.

## Lattice dynamics of Mg(OH)_2_

**Figure 2:**
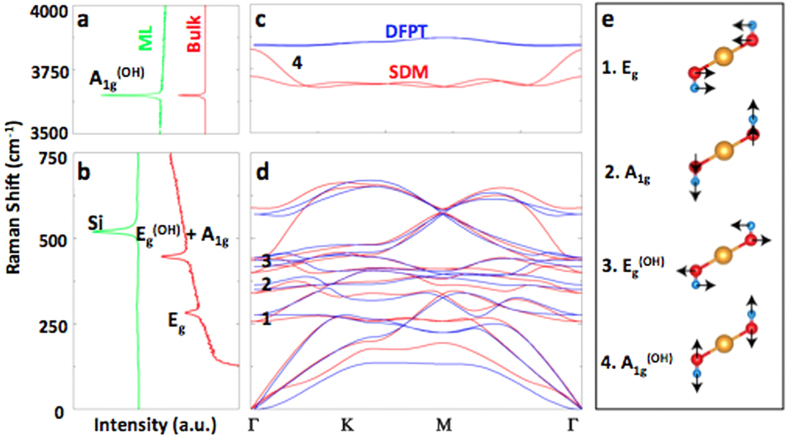
Phonon dispersion and Raman spectrum of Mg(OH)_2_. Raman spectroscopy measurements taken from thick flakes and monolayers of Mg(OH)_2_ in the (**a)** high frequency (3500–4000 cm^−1^) and (**b)** low frequency (0–750 cm^−1^) range. (**c-d)** Phonon dispersion calculated by density functional perturbation theory (DFPT) in blue and small displacement method (SDM) in red for high and low frequencies, respectively. (**e)** Corresponding optical modes highlighted in Fig. 2a,b.

The unit cell of the Mg(OH)_2_ contains five atoms (Fig. 1a); therefore, the phonon dispersion yields three acoustic and 12 optical modes where the vibration representation of the optical modes at zone center is described as Γ = 4E_u_ + 2A_2u_ + 4E_g_ + 2A_1g_^21^. These modes for monolayer Mg(OH)_2_ are calculated by small displacement method (SDM) and density functional perturbation technique (DFPT), as shown in Fig. 2c,d (red and blue lines), and compared to the Raman spectrum of monolayer as well as bulk Mg(OH)_2_ flakes in Fig. 2a,b. In the back scattering configuration, like in our spectrometer, only E_g_ and A_1g_ modes (Fig. 2e) can be observed experimentally. E_g_ and A_1g_ modes, shown in Fig. 2e, have the translational motion, meaning O-H bond distance is mostly fixed, whereas E_g_^OH^ and A_1g_^OH^ modes correspond to the librational vibration of O and H atoms where O-H bond distance changes. Modes indexed with (OH) are undefined for TMDs, e.g. MoS_2_ and MoSe_2_, as the –OH groups are replaced by chalcogens and the presence of these modes are the primary difference between Mg(OH)_2_, or AMHs family members, and TMDs^22^.

Raman spectrum of bulk Mg(OH)_2_ displays two prominent peaks located at 280 and 445 cm^−1^ (Fig.2b red solid line), where the first peak is associated with E_g_ mode and the second peak is a combination of (degenerate) of A_1g_ and E_g_^OH^ modes (see Figure S2). As the material is thinned to a monolayer (green solid lines), these peaks completely disappear due to the low optical absorption coefficient of wide band gap Mg(OH)_2_. Consequently, low-frequency Raman peaks offer no substantial information on mono- and few-layers ( < 10 layers), and in fact the material analysis and quantification cannot be performed by usual first order Raman modes (E_g_ and A_g_). On the other hand, A_1g_^OH^ is a breathing mode of the –OH group when the alkaline atom is stationary which involves O and H atom motion in the out-of-plane direction. Since both H and O atoms are light, its peak position appears at very high frequencies (ω ~ 3650 cm^−1^) as shown in Fig. 2a. Unlike E_g_, A_1g_, and E_g_^OH^ modes, A_g_^(OH)^ peak is observed clearly from bulk to monolayers owing to its large Raman scattering cross-section under visible (488 nm) excitation.

## Anomalous dimensionality effects from 3D to 2D

**Figure 3:**
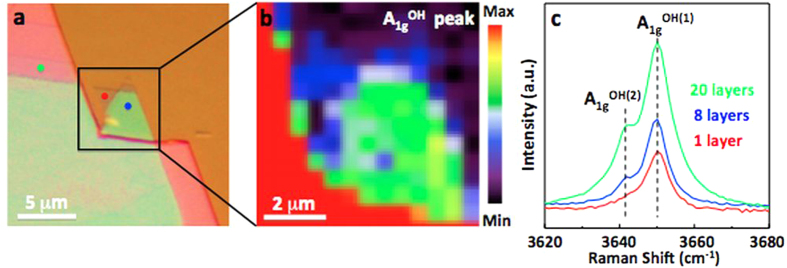
Anomalous Lattice Vibration in 2D Mg(OH)_2_ (**a**) Optical image of mapped Mg(OH)_2_ flakes. (**b)** Actual Raman mapping at 3650 cm^−1^ for A_1g_^OH^ mode. (**c)** Representative Raman spectrum acquired from highlighted thick (green), few-layer (blue), and monolayer (red) regions (see data acquisition points in Fig. 3a).

Presence of A_1g_^OH^ peak for 2D Mg(OH)_2_ is clearly seen from Raman mapping measurements on exfoliated flakes. Mapping at 3650 cm^−1^ shows that A_1g_^OH^ peak intensity gradually decreases from thick to 2D regions due to weak Raman scattering from thinner (lesser) materials, but A_1g_^OH(1)^ signal remains finite for 2D sheets. A closer look at the A_1g_^OH^ mode (Fig. 3c) shows that its peak shape dramatically changes, as flakes are isolated down to 2D. The doublet feature (green and blue curves) observed for bulk and thick flakes transforming into one single peak (red curve) in the 2D limit as shown at the top of Fig. 3c. In this regard, the peak shape of A_g_^(OH)^ provides a quick way to assess the material thickness and presence of 2D Mg(OH)_2_.

To understand the dimensionality effects observed on A_g_^(OH)^ mode, we decomposed A_g_^(OH)^ into two peaks -located at 3650 and 3642 cm^−1^-, and labeled them as A_1g_^OH(1)^ and A_1g_^OH(2)^ for easy reference (Fig. 3c). DFT vibrational spectrum analysis of their eigen-motions shows that these two peaks are associated with in-phase and out-of-phase –OH breathing modes between adjacent layers and estimated to be separated by 10.7 cm^−1^. This closely aligns with the experimentally determined value of Δω~8 cm^−1^. More specifically, if A_1g_^OH^ mode (–OH bonds) in one layer is stretching (bond distance is increasing) and –OH bonds in the adjacent layers are contracting (bond length is decreasing), this mode is called the out-of-phase vibration and labeled as A_1g_^OH(1)^. In this mode, the restoring forces on –OH groups are minimized by geometrical consideration, and thus yield a softer mode at lower frequencies. Similarly, the interaction (restoring force) is greater when the OH breathing motion of all the layers are in-phase, and hence A_1g_^OH(2)^ appears at lower frequencies (by ~8 cm^−1^). Based on this model, after flakes are isolated to 2D, A_1g_^OH(2)^ mode becomes absolute (there is no nearby –OH group acting as a large restoring force on the monolayer), leaving a single Raman peak in the close vicinity of A_1g_^OH(2)^ mode (Fig. 3c red).

## Dimensionality effects on the electronic structure of Mg(OH)_2_

**Figure 4:**
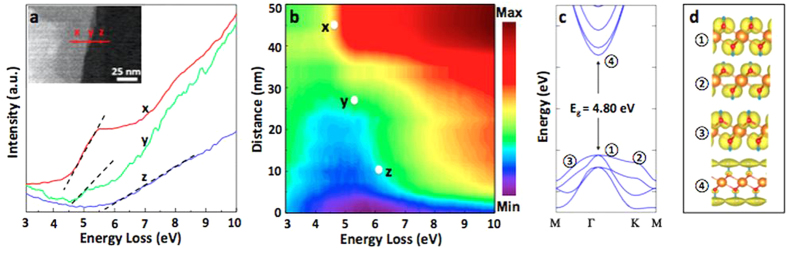
Nano-EELS measurements and band renormalization in 2D Mg(OH)_2_ (**a)** Nano scale Electron Energy Loss Spectra (nano-EELS) on thick- (x), few- (y), and mono- (x) layered Mg(OH)_2_ sheets. (**b)** Nano-EELS mapping on Mg(OH)_2_ thick, few-, and mono- layered flakes. (**c)** Calculated electronic band structure of monolayer Mg(OH)_2._ (**d)** Orbital characters of bands located at some of the high symmetry points highlighted in the calculated band structures.

We performed nm spatial resolution and probing spot electron energy-loss spectroscopy (nano-ELLS) on mono-, few-, and thick- layered Mg(OH)_2_ sheets deposited onto TEM grids to estimate the electronic band gap of the material for different numbers of layers (Fig. 4a). Notice that photoluminescence measurements under 325 nm laser excitation yield zero PL intensity likely due to low optical absorption as well as large non-radiative recombination; and thus, nano-EELS measurements are essential for understanding the effects of dimensionality on the electronic properties of Mg(OH)_2_.

When incoming electron energy closely matches the electronic band gap of the material, electrons are absorbed by the material and lose some of their kinetic energies. At this energy point, the intensity of EELS spectrum sharply increases and extrapolation of this slope to the abscissa (energy) yields the band gap value of the material^23^. Figure 4a shows the EELS spectra collected from thick (point x) to monolayer (point z) segments of the Mg(OH)_2_ flake on a TEM grid (Fig. 4a inset). It can be seen in Fig. 4a that the band gap increases from 4.6–5.5 eV going from point x to point z as a result of band renormalization, confinement effects in 2D insulating materials, and presented nano-EELS measurements mark the first experimental demonstration of band normalization in layered insulating material. Dimensionality-induced shift in the band gap value of Mg(OH)_2_ can be observed from the nano-EELS mapping depicted in Fig. 4b. Observe that these band values are suitably located between semiconducting TMDs^24^,^25^ and insulating h-BN (6 eV or larger)^26^,^27^, expanding the library of available 2D materials.

Theoretically, the electronic band structure of 2D Mg(OH)_2_ along high symmetry points in the first Brillouin zone is shown in Fig. 4c. Based on DFT calculations, monolayer Mg(OH)_2_ is a direct band gap insulator with optical gap values of 4.80 eV (theoretical) and the valance band maximum (VBM) and conduction band minimum (CBM) located at the Γ symmetry point. In Fig. 4d, we show band- and k-point decomposed charge density of band edges at fixed iso-values (isosurface-level is set to 0.0196). Orbital analysis results indicate that the first (VBM at Γ) and third points (VBM at M) involve p_x_ and p_y_ orbitals of O atoms, whereas the second band at (VBM at K) has p_z_ orbital character. The fourth band (CBM at Γ) exhibits a well-delocalized state over the surface, a free-electron-like state, and the charge density which is almost not visible becomes visible with the choice of a very low iso-value.

Observed discrepancy between experimentally and theoretically estimated optical band gap values is anticipated as DFT calculations typically underestimate the band gap values due to lack of proper description of functionals used in calculations. These deficiencies are particularly apparent from DFT calculations on bulk Mg(OH)_2_, predicting larger band gap value compared to monolayers, which conflicts with band renormalization theory within the quantum confinement limit as well as our experimentally determined values (E_g_^Bulk^ < E_g_2D). Nevertheless, calculated band offset values have been summarized in supplementary information S1.

## Surface polarization and Mg(OH)_2_/MoS_2_ heterostructures

**Figure 5:**
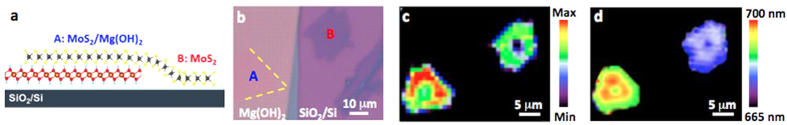
Heterostructure of MoS2/Mg(OH)_2_ (**a)** Schematic presentation of demonstrated MoS_2_/Mg(OH)_2_ heterostructure on SiO_2_/Si substrate. (**b)** Optical images taken from MoS_2_/Mg(OH)_2_ heterostructure (**A**, bule) and pristine MoS_2_ (**B**, red) on SiO_2_/Si substrate. (**c)** Integrated PL intensity and (**d)** PL peak position mapping at the selected area in Fig. 5b.

DFT Bader charge transfer analysis reveals that 0.9 e^−^/atom and 0.5 e^−^/atom charges transfer from Mg to O atoms and H to O atoms respectively. The charge transfer between Mg, O, and H creates a highly polarized (charged) surface which is, in principle, capable of providing (transferring) extra carriers into dissimilar 2D materials, e.g. MoS_2_ and MoSe_2_, when in contact with Mg(OH)_2_ layers in the vertical heterojunction geometry (Fig. 5a). We anticipate that substrate induced self-doping effect provides an efficient way to enhance PL intensity of MoS_2_ by tuning the neutral (eh) to a charged (eeh) exciton population after withdrawing electrons from unintentionally *n*-doped MoS_2_ monolayers, along with creating a more uniform charge density on the MoS_2_ surface.

Based on this self-doping mechanism, we fabricated vertical MoS_2_/Mg(OH)_2_ heterojunctions by transferring CVD grown MoS_2_ monolayers onto exfoliated few-layered Mg(OH)_2_ flakes on SiO_2_/Si substrates, shown as flake A in Fig. 5b. Single point PL measurements reveal that light emission intensity increases considerably after MoS_2_ is transferred onto Mg(OH)_2_. As shown in Fig. 5c, this PL enhancement effect and related PL peak shift can be clearly seen on the MoS_2_/Mg(OH)_2_ region (flake A), where integrated PL intensity increases as much as 100% with a PL peak position change from 1.83 eV (for pristine MoS_2_) to 1.81 eV (Fig. 5d). Similar trends and PL enhancement were observed on more than 20 separately fabricated samples.

The observed changes in the PL peak intensity and peak position can be explicated by the following physical mechanisms. Previously, PL intensity of sTMDs monolayers was modulated for more than an order of magnitude by changing the sheet carrier concentration with various techniques such as inter-molecular gating^28^ and conventional back-gate biasing^28^,^29^. We found that in close proximity to undoped (doped) limit, the light emission is strong (weak) and the emission process is mostly dominated by efficient (inefficient) neutral excitons (trions)^29^. Within this picture, after MoS_2_ is deposited onto few-layer thick Mg(OH)_2_, an electron is transferred from MoS_2_ to Mg(OH)_2_ due to presence of an excess amount of electrons on the MoS_2_ side. These access carriers in MoS_2_ have been previously reported and speculated to originate from chalcogen vacancies and/or unintentional impurities introduced during CVD growth, and immediately transfer to a positively charged Mg(OH)_2_ surface. Consequently, MoS_2_ layers are relatively depleted, forming efficient (luminescent) neutral exciton complexes and enhancing the PL intensity.

Using modified Mg(OH)_2_ crystal growth technique, 2D insulating Mg(OH)_2_ sheets are isolated to monolayers from their bulk counter-parts, allowing to study its vibrational and electronic properties of 2D Mg(OH)_2_ for the first time. We find that its vibrational properties are vastly different from other 2D materials known to date. Raman spectroscopy and vibration analysis reveal that hydroxyl group out-of-plane breathing mode located at high frequencies (~3650 cm^−1^) dominates the lattice modes, which dictates the dimensionality effects and provides an effective way to characterize 2D Mg(OH)_2_ sheets. In addition, nano-EELS measurements allowed us to link electronic properties to the number of layers for the first time on 2D insulating materials, pointing towards unusual quantum confinement effects in Mg(OH)_2_. Heterostructures of CVD MoS_2_ monolayers on highly polar Mg(OH)_2_ facilitate a large charge transfer at the Mg(OH)_2_–MoS_2_ interface, providing effective means to manipulate excitonic dynamics. Results not only establish unusual confinement effects in 2D Mg(OH)_2,_ but also offer novel 2D insulating material with unique physical, vibrational, and chemical properties for potential applications in flexible optoelectronics.

## Methods

### CVD growth

MoS_2_ monolayers were produced by CVD technique. Si with 300 nm SiO_2_ was used as a growth substrate. The substrates were cleaned in piranha solution (H_2_SO_4_:H_2_O_2_;3:1) and then they were placed with face up above on to the top of alumina boat which was containing 3.5 mg MoO_3_ (≥99.5% Sigma Aldrich). The ceramic boat was placed to the center of the heating zone of the furnace. Another alumina crucible containing sulphur (≥99.88% Sigma Aldrich) was placed in to the tube in a gas flow direction. The distance between substrates and sulphur containing boat was 23 cm. the monolayer growth was performed under atmospheric pressure with high purity nitrogen. The furnace heated to 575 °C with 140 s.c.c.m. and then flow rate was decreased to 40 s.c.c.m. After melting of the sulphur at 665 °C, flow rate was decreased to 5 s.c.c.m. and then the temperature kept at 690 °C with 5 s.c.c.m. for 10 minutes. Samples cooled down to the 500 °C at a rate of 21 °C min^−1^ with 40 s.c.c.m. When the temperature was 500 °C , the furnace opened and samples cooled down to the room temperature quickly.

### Crystal growth

AMHs crystals were synthesized using hydrothermal growth routes by two different precursor reactants. The first technique was based on reaction between alkaline metal nitrates, Mg(NO_3_)_2_ Here, purified Mg(NO_3_)_2_ solution was placed in a autoclave chamber and was heated to 125 °C for 6 hours and then NH_3_ was introduced into the autoclave and kept at the same temperature for 1–3 days. The reaction chamber was slowly cooled down to room temperature and the materials were separated from the reaction solution by standard filtration to be dried at 105 °C in vacuum. In the second technique Mg(NO_3_)_2_ (0.05 M) and N_2_H_4_·H_2_O was mixed to form precipitation and rest of the reaction occured in teflon lined stainless steel autoclave at 165 °C for 1–3 days. Grown materials were centrifuged, filtered, washed with distilled water, and dried in vacuum at 100 °C. Synthesized crystals were around couple Teflon-lined stainless steel autoclave lamellar behavior with transparent look. XRD measurements on the Mg(OH)_2_ displayed sharp 0001 peak that correspond to 4.86 A interlayer spacing values which is in close agreement with the c-axis values estimated from our DFT calculations (Figure S1).

### Heterostructure fabrication

The heterostructure was fabricated by transferring CVD-grown MoS_2_ monolayers (described above) onto exfoliated Mg(OH)_2_ flakes on SiO_2_/Si substrate. The monolayer MoS_2_ flakes were first covered by Poly(dimethyl siloxane) (PDMS, Sylgard 184, Dow Corning) film which were prepared by mixing its precursors followed by heat curing at 75 °C for 30 mins. Then, the MoS_2_ single-layers were transferred onto PDMS from SiO_2_/Si substrate by heated KOH (1 mol/L) etching at 60 °C The KOH residue was further removed by cleaning the detached PDMS/MoS_2_ film in deionized water. After dried by nitrogen flow, the film was finally adhered onto Mg(OH)_2_ thin flakes for 30 mins. before peeled off. Note, the MoS_2_/Mg(OH)_2_ heterostructure can be successfully prepared using the above method due to the highly polarized Mg(OH)_2_ surface, as well as the lower surface energy of SiO_2_/Si substrate

### Spectroscopy and optical measurements

The photoluminescence and Raman measurements were performed in a Renishaw InVia Raman microscope by using a laser excitation source of wavelength 488 nm under 100× objective lens (0.95 NA). Laser power was set to 1 mW on 10 mm^2^ area.

### Nano-EELS measurements

(Valence electron energy loss spectroscopy) was performed using Nion High-Resolution Monochromated EELS STEM (HERMES) system consisting of a Nion STEM100 scanning transmission electron microscope (STEM) equipped with a Nion high energy resolution monochromator and a modified Gatan Enfinium EEL spectrometer. The energy resolution, measured from the half width of the zeroloss peak was set to be 60 meV, to improve S/N, and the accelerating voltage was 60 kV, the probe size was about 0.3 nm, and the probe current was approximately 12 pA .

### Theoretical calculations

The calculations were performed using the Vienna ab initio simulation package (VASP)^30^,^31^. The core-valence interaction was described by the frozen-core projector augmented wave (PAW) method^32^. Both the generalized gradient approximation of Perdew-Burke-Ernzerhof (GGA-PBE)^33^ and the Heyd-Scuseria-Ernzerhof (HSE06) hybrid functional^34^ were adopted for exchange-correlation functional. Energy cut off for plane-wave expansion was set to 500 eV. The Brillouin zone was sampled by 12 × 12 × 1 (12 × 12 × 9 for bulk) Monkhorst-Pack (MP) special k point meshes. A vacuum layer larger than 10 Å was added to avoid interaction between adjacent images. Structure relaxation was stopped when the force on each atom was smaller than 0.01 eV/Å. For MoS2, spin-orbit interaction was taken into account when calculating band structures. The vacuum level was taken as zero reference in the calculations of band alignment. For the calculation of phonon dispersions we utilize both small displacement (SD) and density functional perturbation theory (DFPT) methodology^35^,^36^. While DFPT calculations performed using the primitive unitcell of metal hydroxides, in SD calculations for the convergence of dynamical matrix we use 5 × 5 × 1 supercell.

## Additional Information

**How to cite this article**: Suslu, A. *et al*. Unusual dimensionality effects and surface charge density in 2D Mg(OH)_2_. *Sci. Rep*. **6**, 20525; doi: 10.1038/srep20525 (2016).

## Supplementary Material

Supplementary Information
