# Supplementary material for: Unusual dimensionality effects and surface charge density in 2D Mg(OH)2
Source: Sci Rep. 2016 Feb 5;6:20525. doi: 10.1038/srep20525 (PMC4742812; doi:10.1038/srep20525)
Supplement: Supplementary Information [file srep20525-s1.pdf]

Unusual dimensionality effects and surface charge density in 2D $Mg(OH)_2$

Aslihan Suslu[1+], Kedi Wu[1+], Hasan Sahin[3+], Bin Chen[1], Sijie Yang[1], Hui Cai[1], Toshihiro Aoki[1,2], Seyda Horzum[3], Jun Kang[3], Francois M. Peeters[3], and Sefaattin Tongay[1]*

[1] School for Engineering of Matter, Transport and Energy, Arizona State University, Tempe, AZ, 85287, USA

[2] LeRoy Eyring Center for Solid State Science, Arizona State University, Tempe, AZ 85287, USA

[3] Department of Physics, University of Antwerp, Campus Groenenborger, Groenenborgerlaan 171, 2020, Antwerp, Belgium

Supplementary Information

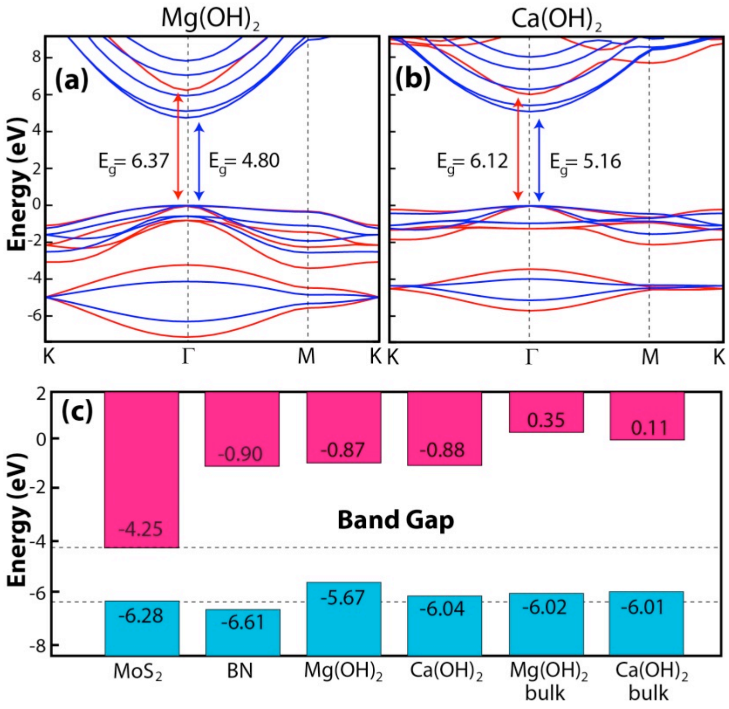

Figure S1. (**a**) Band structure of monolayer $Mg(OH)_2$ and $Ca(OH)_2$ calculated by PBE (**b**) The absolute

band alignment of MoS_2, BN, monolayer and bulk $Mg(OH)_2$ and $Ca(OH)_2$ calculated by PBE. The vacuum level is set as zero energy. Band dispersion of single layer and bulk structures are shown by blue and red lines, respectively. **(c)** The absolute band alignment of MoS_2, BN, monolayer $Mg(OH)_2$ and $Ca(OH)_2$ calculated by HSE06. The vacuum level is set as zero energy.

Single layer crystal structures of AMHs of Mg and Ca belong to the space group of D^3_{3d}. In the optimized structure a lattice parameter of hexagonal primitive unit cell of $Mg(OH)_2$ and $Ca(OH)_2$ are 3.18 and 3.62 Angstrom, respectively. In addition, for the bulk structures of $Mg(OH)_2$ and $Ca(OH)_2$ a- c lattice parameters are calculated to be 3.14-4.62 and 3.56-4.74, respectively. The band structures of bulk and monolayer $Mg(OH)_2$ and $Ca(OH)_2$ obtained by HSE06 are shown in Fig S1(a) and S1(b). It is seen that both single layer and bulk hydroxides of Mg and Ca exhibit direct band gaps at the Gamma point. The band dispersion obtained by PBE and HSE06 are very similar, except for the band gap values. Usually the band gaps of semiconductors are severely under estimated by PBE. Inclusion of HSE06 correction, combining the short-range Fock exchange and semi-local long-range exchange, results in more accurate band gap values. For monolayers, the band gaps are 4.80 (3.30) eV for $Mg(OH)_2$ and 5.16 (3.68) eV for $Ca(OH)_2$ from HSE06 (PBE). For bulk materials, the band gaps are 6.37 (4.59) eV for $Mg(OH)_2$ and 6.12 (4.37) eV for $Ca(OH)_2$ from HSE06 (PBE). The valence bands of monolayer $Mg(OH)_2$ and $Ca(OH)_2$ consist of the upper part (-4eV ~0 eV) and the lower part (-6 eV~-4 eV). The upper part is mainly contributed by the O px+py orbitals, and the lower part is contributed by the O-pz and H-s orbitals. It is also notable that the conduction bands of these monolayers are parabolic, indicating that these states are free-electron like. Fig. S1(c) also presents the absolute band alignment of MoS_2, BN, monolayer and bulk $Mg(OH)_2$ and $Ca(OH)_2$ calculated by HSE06. MoS_2 forms type-I alignment with BN and type-II alignment with other materials. The CBM of BN, monolayer $Mg(OH)_2$ and $Ca(OH)_2$ are very close to each other. The difference in their band gap mainly affects the valence band offset. Compared with monolayers, bulk $Mg(OH)_2$ and $Ca(OH)_2$ have larger band gap. The VBM of bulk $Mg(OH)_2$ is lower than that of monolayer, and its CBM is higher. For $Ca(OH)_2$, the VBM of bulk and monolayer are quite close,

but the CBM of bulk is much higher.

Supplementary Figure 2

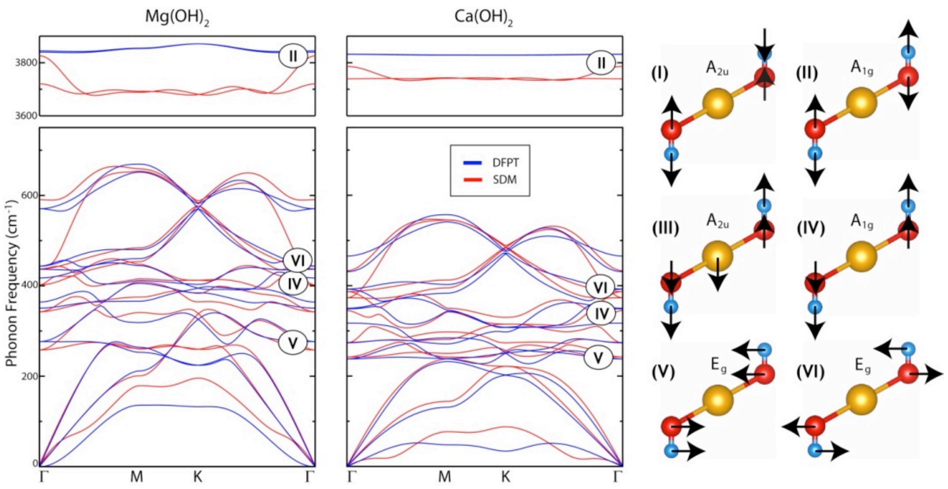

Figure S2. Calculated phonon dispersion of single layer hydroxides of Mg and Ca. For the sake of comparison both results of SDM and DFPT are presented. Eigenmotions of various Eg and Au phonon vibrations are illustrated in the right panel.

Although the electronic properties of single layer metal hydroxides are well approximated by DFT calculations, ionic nature of the bonds makes the calculation of vibrational characteristics of these structures harder than covalent materials. To point out the possible differences between various computational techniques we further employ phonon calculations using Density Functional Perturbation Theory (DFPT). Differing from Small Displacement Method that requires the use of large supercells for the convergence of elements of dynamical matrix, in DFPT methodology calculation of the ground state charge density and its response to distortion of nuclear geometry using primitive unitcell is enough. In Figure S2, using both approaches, we present phonon dispersion of single layer $Mg(OH)_2$ and $Ca(OH)_2$. It is seen that the high-energy OH modes are more dispersive in SDM whereas the quadratic behavior of flexural modes are better approximated by DFPT theory. These results show that depending on whether

the phonon branch has very low or very high frequency various approaches can be favorable over other one. However, in mid-frequency region both methods are in good agreement.
